# Socioeconomic status and the prevalence of fever in children under age five: evidence from four sub-Saharan African countries

**DOI:** 10.1186/1756-0500-5-380

**Published:** 2012-07-27

**Authors:** Jacob Novignon, Justice Nonvignon

**Affiliations:** 1 Department of Economics, University of Ibadan, Ibadan, Nigeria; 2 Department of Health Policy, Planning & Management, School of Public Health, University of Ghana, Legon, Ghana

**Keywords:** Fever, Children under age five, Sub-Sahara Africa

## Abstract

**Background:**

The burden of fevers remains enormous in sub-Saharan Africa. While several efforts at reducing the burden of fevers have been made at the macro level, the relationship between socioeconomic status and fever prevalence has been inconclusive at the household and individual levels. The purpose of this study was to examine how individual and household socioeconomic status influences the prevalence of fever among children under age five in four sub-Saharan African countries.

**Methods:**

The study used data from the 2008 Demographic and Health Survey (DHS) from Ghana, Nigeria, Kenya and Sierra Leone with a total of 38,990 children below age five. A multi-level random effects logistic model was fitted to examine the socioeconomic factors that influence the prevalence of fever in the two weeks preceding the survey. Data from the four countries were also combined to estimate this relationship, after country-specific analysis.

**Results:**

The results show that children from wealthier households reported lower prevalence of fever in Ghana, Nigeria and Kenya. Result from the combined dataset shows that children from wealthier households were less likely to report fever. In general, vaccination against fever-related diseases and the use of improved toilet facility reduces fever prevalence. The use of bed nets by children and mothers did not show consistent relationship across the countries.

**Conclusion:**

Poverty does not only influence prevalence of fever at the macro level as shown in other studies but also the individual and household levels. Policies directed towards preventing childhood fevers should take a close account of issues of poverty alleviation. There is also the need to ensure that prevention and treatment mechanisms directed towards fever related diseases (such as malaria, pneumonia, measles, diarrhoea, polio, tuberculosis etc.) are accessible and effectively used.

## Background

In sub-Saharan Africa (SSA), fever remains a major public health problem. Fever is a symptom of diseases especially predominant among children under age five [1]. Such diseases include malaria, diarrhoea, pneumonia, measles, polio and tuberculosis, to mention a few. These diseases are main contributors to deaths among children under age five in the sub-Saharan African region [2-4]. The World Health Organization’s (WHO) report on infectious diseases posits that these diseases cause most of the deaths from infectious diseases [5].

The burden of fever is reflected in the burden of diseases with fever as a major symptom as mentioned above. Black et al. [6] estimate that infectious diseases caused 68% out of all deaths among children under age five. Pneumonia contributed about 18% of infectious deaths followed by Diarrhoea (15%), Malaria (8%) and Measles (1%) [6]. Table 1 shows estimated numbers of deaths by cause in children under age five in Ghana, Nigeria, Kenya and Sierra Leone. The table suggests that fever related diseases have enormous mortality burden in SSA.

**Table 1:** Estimated numbers of deaths by cause in children under age five

**Country**	**Diarrhoea**	**Measles**	**Malaria**	**Pneumonia**
Ghana	5193	1115	14365	5712
Kenya	38802	1568	20666	30406
Nigeria	201368	707	217357	177212
Sierra Leone	8904	2027	5520	8444

Source: Adapted from Black et al (2010).

There are effective medicines and control strategies to reduce the prevalence of fever, especially among children and the WHO has made several efforts to ensure effective treatment of fever-related diseases [2,5]. However, the impact of these strategies in developing regions is typically very little or not present at all. This can be attributed to lack of funding to implement strategies, lack of government commitment and poor health care systems [5]. While these macro level factors may be genuine, a critical understanding of the factors that influence fever prevalence at the micro level will be important for policy intervention.

The socioeconomic status and fever nexus is multifaceted and complicated. However, a comprehensive analysis of this relationship across individual characteristics has received little attention [7]. Some empirical evidence shows that better socioeconomic status relates to lower incidence of fever compared to worse-off socioeconomic status [8-11]. On the contrary, a reverse relationship between socioeconomic status and fever prevalence have been established by other studies [12]. Nonvignon et al. [13] show that, at the individual level, socioeconomic status influences fever prevalence through treatment choices, as the wealthy are more likely to seek early and effective treatment relative to the poor.

While the burden of fever in poor countries and regions has been well researched with unanimous conclusions, the specific socioeconomic relationship that exists at the individual level has been inconclusive [9,14,15]. The purpose of the current study is, therefore, to examine the relationship between socioeconomic status (i.e. wealth status) and the prevalence of fever in children under age five across selected countries in sub-Saharan Africa.

## Methods

### Data

Data on children under age five from the 2008 Demographic and Health Survey (DHS) from four sub-Sahara African countries (i.e. Ghana, Nigeria, Kenya and Sierra Leone) were used for the study. The sample includes a total of 2,794 children under age five in Ghana, 25,447 in Nigeria, 5,706 in Kenya and 5,043 in Sierra Leone [16-19]. The selection of countries was based on the availability of DHS data for the year 2008 which also allowed for easy data pooling. Moreover, in these regions, fevers are major symptoms of such diseases as malaria, pneumonia, measles and diarrhoea which are major health concerns as shown in Table 1[1,3,20].

The DHS is a nationwide household-based survey implemented in a representative probability sample. The sample design was at two stages; first, sample points or clusters were selected from a master sampling frame (constructed from a national population and housing census). The clusters were selected using systematic sampling with probability proportional to size. To provide a sampling frame for the second stage selection of households, a complete household listing operation was conducted in all the selected clusters. The second stage, therefore, involved a systematic sampling of households listed in each cluster. This was to ensure adequate numbers of completed individual interviews to provide estimates for key indicators with precision. [21-24].

### Variables and outcome of interest

Reported prevalence of fever in children under age five during the two weeks preceding the survey was used as the outcome variable. The variable is a dummy which takes on the value of 1, if a child had fever and 0, otherwise. The two-week period reduces possible recall bias that may arise. However, the measure was self reported, hence, likely to contain some bias [25,26].

The main explanatory variable of interest was the household socioeconomic status which was measured by the wealth index quintile computed from household asset index. The index was computed from household data on ownership of consumer goods and characteristics of dwelling place including socioeconomic assets such as car, television, radio, gas cooker, electricity etc.. Each of these assets were assigned weights (factor score) which were then standardized to generate the index [7]. The wealth index quintiles were the most appropriate measure of household socioeconomic status in the DHS dataset.

Other explanatory variables used in the study include specific age of child, sex, use of bed nets, availability of improved source of drinking water and toilet facility and health insurance coverage. Vaccination against Polio, Measles and Tuberculosis were also included in the model. Dummy variables for each of these diseases were included and took the value of 1 if the child is vaccinated against the particular disease or 0 otherwise. With the exception of age of child which was continues, sex, use of bed nets, availability of improved source of drinking water and toilet facility and health insurance coverage were also represented as dummy variables with the value of 1 if the response was yes and 0 otherwise. The relationship between socioeconomic status and the prevalence of fever was expected to be negative. That is, children from wealthier households were expected to have lower fever prevalence relative to children from poorer households. Similar results were expected for all countries included in the survey.

### Estimation process

The dependent variable was a binary response variable which takes on the value of 1 if a child was reported to have fever and 0 otherwise, hence, a logistic model was used to examine the effects of socioeconomic status on the incidence of fever among children under age five.

Following Yusuf [7], a random effect logistic model was fitted with both fixed effects and random effects included. The significance of this is that, it allows accounting for the nested nature of the data: children under age five nested within regions/clusters. All the four countries included in the study have geopolitical zones with each of these zones having peculiar idiosyncratic characteristics which include the prevalence of fever. Hence accounting for these variations in the model was important.

Such multi-level analysis makes it possible to consider which cluster/region a child belongs to in estimating the relationship between independent and dependent variables. Using the GLLAMM routine in STATA, the correlation between children from the same cluster or region arises from their sharing specific but unobserved properties of the respective regions. The following random effect logistic regression model for the data was employed:

Logit$p(y_{\mathrm{ij}}=1|x_{\mathrm{ij}},U_{i})=x_{\mathrm{ij}}\beta +U_{i}$

(1) $$U_{i}∼N(0,\tau^{2})$$

Where β represents a vector of fixed effects and U_i_ represents a random effect. The random effect variable has a mean of zero and constant variance. It is also an estimation of the variance across all of the regions involved in the study. The larger the variance, the more dependent is the outcome of interest on the region. Similarly, smaller variance implies that variations in the outcome of interest may only be explained by the measured characteristics.

The value of U_i_ shows the mutual dependence that exists in responses from the same regions. This is to say that the correlation between children from the same regions is completely explained by them having been observed in the same regions. The variance measures the degree of heterogeneity in the probability of experiencing fever that cannot be explained by the classification of whether a child has fever or not.

Such dependences are well described by the intra-class correlation coefficient (ICC) which measures the extent to which individuals within the same group are more similar to each other than individuals in different groups. The following formula was used in calculating the ICC (λ):

(2) $$\lambda =\frac{\delta }{\delta +\pi^{2}/3}$$

Where δ is the estimated variance and π = 3.142.

The GLLAMM approach fits generalized linear models, a class of regression models for univariate responses with density from an exponential family. Using STATA the logistic regression model was specified from the family of generalized linear model with the logit link and binomial distribution using the link ( ) and family ( ) options. The i(region) option was included to account for the desired within region correlation matrix [7,27]. While the GLLAMM approach was used for the country-specific analysis, a normal logistic model was fitted for the combined country data.

## Results

### Descriptive statistics

Table 2 shows that Nigeria had the lowest fever prevalence of about 16%, followed by Ghana with about 20% and Kenya, 25%. Sierra Leone had the highest prevalence of fever among children under age five (26%), marginally higher than that of Kenya. In the combined sample, fever prevalence was about 19%.

**Table 2:** Descriptive statistics

**Characteristics**	**Ghana**	**Nigeria**	**Kenya**	**Sierra leone**	**All countries**
Fever occurrence in child <5 yrs	551 (19.96)	3965 (15.86)	1385 (24.62)	1271 (26.56)	7172 (18.79)
**Wealth index**					
poorest	901 (32.25)	6602 (25.94)	1668 (29.23)	1090 (21.61)	10261 (26.32)
poorer	621(22.23)	5994(23.55)	1007 (17.65)	970 (19.35)	8598 (22.05)
middle	466 (16.68)	4973 (19.54)	912 (15.98)	1043 (20.68)	7394 (18.96)
richer	470 (16.82)	4318 (16.97))	938 (16.44)	1030 (20.42)	6756 (17.33)
richest	336 (12.03)	3560 (13.99)	1181 (20.70)	904 (17.93)	5981 (15.34)
Child age	1.9187	1.9205	1.9439	1.8192	1.9107
**Child sex**					
Male	1420 (50.82)	12873 (50.59)	2923 (51.23)	2552 (50.60)	19768 (50.70)
Female	1374 (49.18)	12574 (49.41)	2783 (48.77)	2491 (49.40)	19222 (49.30
Household posses bed nets	2059 (73.69)	6327 (75.11)	4336 (75.99)	2490 (49.44)	15212 (39.05)
Child <5 yrs slept under bed net	1386 (50.71)	3716 (15.01)	3473 (62.08)	1870 (38.37)	10475 (27.45)
Mother slept under bed net	1337 (47.85)	3381 (13.29)	3350 (58.71)	1908 (37.83)	9976 (25.59)
**Type of bed net**					
Treated	804 (29.90)	1389 (5.69)	2864 (52.19)	1406 (30.41)	6463 (17.37)
Untreated	417 (15.51)	1730 (7.09)	217 (3.95)	113 (2.44)	2477 (6.66)
None	1457 (54.59)	21292 (87.22)	2407 (43.86)	3105 (67.15)	28272 (75.98)
Health insurance	1130 (40.52)	327 (1.29)	288(5.05)	89 (1.78)	1834 (4.73)
**Educational attainment**					
None	1046 (37.48)	12552 (49.33)	1220 (21.38)	3709 (73.55)	18527 (47.52)
Primary	669 (23.97)	5831 (22.91)	3208 (56.22)	652 (12.93)	10360 (26.57)
Secondary	1012 (36.26)	5785 (22.73)	963 (16.88)	613 (12.16)	8373 (21.48)
Tertiary and above	64 (2.29)	1279 (5.03)	315 (5.52)	69 (1.37)	1727 (4.43)
**Place of Residence**					
Rural	1861 (66.61)	18467 (72.57)	4320 (75.71)	3353 (66.49)	28001 (71.82)
Urban	933 (33.39)	6980 (27.43)	1386 (24.29)	1690 (33.51)	10989 (28.18)

In Ghana, 12% of the respondents interviewed were in the highest wealth index quintile while about 32% were in the lowest quintile. Similarly, 14% of respondents in Nigeria were in the highest quintile relative to about 26% in the lowest quintiles. In Kenya, about 21% and 29% of respondents were in the richest and poorest wealth index quintiles, respectively. Sierra Leone had approximately 18% of respondents in the richest wealth quintile and about 22% in the poorest quintile. About 15% of households in the combined sample made up the highest wealth quintile relative to 26% in the lowest quintile (Table 2).

Further, between 74% and 75% of households in Ghana, Nigeria and Kenya had bed nets compared to 49% in Sierra Leone. However, the numbers of children under five who actually slept under bed nets were 15% (Nigeria), 38% (Sierra Leone), 50% (Ghana) and 62% (Kenya). Bed net possession in the combined sample was 39% (Table 2).

### Determinants of childhood fever

The results show that, relative to the lowest wealth index quintile, children in the highest quintile were less likely to report fever in Ghana with an odds ratio (OR) of 0.62. A similar result was found for Nigeria (OR = 0.87) and Kenya (OR = 1.4). In the case of Sierra Leone, the relationship between wealth index quintiles and fever prevalence did not show statistical significance. The combined sample shows that children from the highest quintile were less likely to report fever (OR = 0.6) (Table 3).

**Table 3:** Multi-level logistic model analysis for determinants of fever among children under age five

**Variable**	**Ghana**	**Nigeria**	**Kenya**	**Sierra leon**	**All countries**
**Wealth index**					
Poorer	1.09	1.09**	0.88	0.97	0.95
	(0.83-1.42)	(1.01-1.19)	(0.72-1.07)	(0.80-1.14)	(0.86-1.04)
Middle	1.38**	1.17***	1.19*	1.05	0.88**
	(1.02-1.85)	(1.06-1.28)	(0.97-1.45)	(0.88-1.25)	(0.79-0.98)
Richer	1.17	0.96	1.41***	1.14	0.8***
	(0.84-1.64)	(0.86-1.07)	(1.15-1.74)	(0.95-1.38)	(0.71-0.90)
Richest	0.62**	0.87*	1.4***	1.07	0.6***
	(0.40-0.98)	(0.76-1.00)	(1.11-1.75)	(0.85-1.36)	(0.52-0.69)
Child age	0.66***	0.93**	0.98	1.05	0.66***
	(0.51-0.83)	(0.87-1.00)	(0.85-1.14)	(0.92-1.19)	(0.60-0.72)
Child slept under bed net	1.38*	0.81***	1.32***	1.23**	0.8**
	(0.99-1.93)	(0.70-0.94)	(1.07-1.63)	(1.02-1.19)	(0.67-0.97)
Mother slept	0.66**	1.13	0.71***	0.81**	1.23**
	(0.47-0.92)	(0.97-1.31)	(0.58-0.87)	(0.68-0.98)	(1.02-1.49)
Health insurance coverage	0.98	1	0.54***	0.72	1.33*
	(0.80-1.19)	(0.73-1.37)	(0.39-0.74)	(0.47-1.09)	(0.98-1.82)
Child sex	1.3***	1.01	1.05	1.06	1.08**
	(1.09-1.56)	(0.96-1.08)	(0.94-1.18)	(0.96-1.18)	(1.00-1.15)
Mother’s age square	1	1	1***	1***	1
	(1.00-1.00)	(1.00-1.00)	(1.00-1.00)	(1.00-1.00)	(1.00-1.00)
Square root of child age	3.41***	1.71***	1.13	1.42**	2.22***
	(1.98-5.89)	(1.47-1.98)	(0.82-1.57)	(1.08-1.87)	(1.88-2.63)
Water	1.24	1.04	0.97	1.03	1
	(0.95-1.61)	(0.97-1.11)	(0.85-1.11)	(0.91-1.17)	(0.92-1.08)
Toilet	1.01	1.14***	0.59***	1	1.28***
	(1.78-1.30)	(1.06-1.22)	(0.50-0.70)	(0.88-1.15)	(1.19-1.39)
Tuberculosis vaccination	0.63**	0.98	0.64***	0.47***	1.22***
	(0.41-0.96)	(0.89-1.08)	(0.48-0.85)	(0.38-0.58)	(1.11-1.35)
Polio vaccination	1.09	0.77***	1.14	0.99	0.95
	(0.69-1.71)	(0.72-0.84)	(0.87-1.49)	(0.81-1.21)	(0.87-1.04)
Measles Vaccination	1.16	0.76***	1.16	0.75***	0.93
	(0.83-1.61)	(0.69-0.83)	(0.96-1.41)	(0.64-0.88)	(0.85-1.02)
					
Variance	0.05	0.25	0.11	0.22	
Standard Deviation	0.03	0.06	0.06	0.09	
ICC	0.01	0.07	0.03	0.06	

Note: 1. 95% confidence interval are reported in parenthesis.

2. ***, **, * show significance at 1%, 5% and 10% respectively.

Whilst children whose mothers slept under bed nets were less likely to report fever in Ghana (OR = 0.66), Kenya (OR = 0.71) and Sierra Leone (OR = 0.81), the reverse was established in Nigeria but the relationship was not statistically significant (Table 3). Children under five who slept under bed nets were more likely to report fever in Ghana (OR = 1.38), Kenya (OR = 1.32) and Sierra Leone (OR = 1.23). In the case of Nigeria, children under five who slept under bed nets were less likely to report fever (OR = 0.81). Moreover, the combined sample suggest that the use of bed nets by children under five significantly reduces their chances of reporting fever (OR = 0.80) (Table 3).

While the availability of improved water source and toilet facility did not show any statistical significance in Ghana and Sierra Leone, children from households with improved toilet facility were less likely to report fever in Kenya (OR = 0.59). The combined sample suggest that the use of improved toilet facility relates with higher prevalence of fever (OR = 1.28). A similar relationship was established in the case of Nigeria. The availability of improved water source was not significant across all countries and in the combined sample (Table 3).

Vaccination against Tuberculosis (BCG) significantly reduces fever prevalence in Ghana (OR = 0.63), Kenya (OR = 0.64) and Sierra Leone (OR = 0.47). While vaccination against Measles was significant in Nigeria (OR = 0.76) and Sierra Leone (OR = 0.75), Vaccination against Polio was only significant in Nigeria (OR = 0.77) (Table 3).

The estimated between-region variance was 0.05 for Ghana (SE = 0.03; ICC = 0.01), 0.25 for Nigeria (SE=0.06; ICC=0.07), 0.11 for Kenya (SE = 0.06; ICC = 0.03) and 0.22 for Sierra Leone (SE = 0.09; ICC = 0.06) (Table 3).

## Discussions

Generally, the results suggest that children in the highest wealth index quintile were less likely to report fever relative to children in the lowest quintile. This relationship was significant and consistent across all the countries with the exception of Sierra Leone which did not show any statistical significance. The result was also confirmed by the combined sample. This finding implies that poverty plays a critical role in the prevalence of fever in developing regions such as sub-Saharan Africa. This relationship is confirmed by other studies [28-31]. For instance Njau [32] found that people from better-off households were less likely to report episodes of fever than those from worse-off households. One reason that could account for this relationship is that wealthier households are better placed to provide preventive measures to household members including children under age five. Additionally, in the event of fever occurrence, financial access to early and quality health care services is easier among wealthy households. Moreover, in sub-Saharan African countries where health care costs are high, poverty could be a major barrier to seeking early and effective treatment.

The statistically insignificant relationship between wealth status and fever prevalence, in the case of Sierra Leone, was surprising as the rich were expected to have lower incidence of fever. While this relationship is difficult to justify, it can be speculated that the country’s decimated health system (due to the civil war) has made access to early and effective health care scarce for both the rich and the poor.

In Ghana, where social health insurance has in the past few years provided financial access to poor households, one would expect the relationship between the prevalence of fever and poverty to be a weak one. However, it must be noted that at the time of the data collection, the National Health Insurance Scheme (NHIS) in Ghana had been operational for only three years. The relationship between prevalence of fever and poverty may be different currently, when about 66.4% of individuals are registered with the NHIS [33].

Further, the relationship between bed net usage and prevalence of fever by children was not consistent across countries. While the relationship was positive in Ghana, Kenya and Sierra Leone, it was negative in Nigeria and in the combined sample. The latter relationship was expected as the use of bed net is considered to be an important step to reducing the burden of fever in sub-Saharan Africa [34,35]. This finding does not conform with the finding by Yusuf [7] that there is no association between bed net use and fever prevalence among children under five in Nigeria. Much as the finding in the case of Ghana, Kenya and Sierra Leone was unexpected and difficult to explain, factors such as the level of use of the nets (e.g. frequency of use and effectiveness) and whether or not the nets were re-treated with the appropriate insecticides may account for these findings. Krezanoski et al.. [36] argue that households do not effectively use bed nets, hence, poor outcomes in terms of prevention and mortality.

Nevertheless, it is worth noting that children under five whose mothers slept under bed nets were less likely to report fever in Ghana. This finding is intuitively appealing following the speculation that mothers who sleep under bed nets are expected to have good knowledge and understanding of prevalence of fever. Such mothers can therefore be expected to easily influence the appropriate use of bed nets by their children. A similar result was also found by Killeen [37] who posits that the relationship may be explained partially by the spatial effects of ITNs.

Vaccination against Measles, Polio and Tuberculosis remains critical to the prevalence of fever through the control of these diseases. The result suggests that children under age five vaccinated against fever-related diseases were less likely to report fever. This relationship does not come as much surprise as vaccination against these diseases has been well embraced and practiced across several health systems in developing countries. This also shows consistency with literature that effective vaccination has positive effect on fever prevalence [38].

The study was limited by the use of a self-reported measure of fever which could have some bias. Also, while a more specific measure of welfare (i.e. household income and expenditure data) would be appropriate for a study like this, such information was not available in the DHS dataset. The wealth index quintile, therefore, was used as a proxy for socioeconomic status. The study is also limited by its inability to control for seasonality for the occurrence of malaria. Malaria is however just one of the numerous fever-related diseases and hence such seasonality effects may be minimal. The pooled regression results should be interpreted with caution due to the unequal sample size from the countries.

## Conclusion

The study set out to estimate the relationship that exists between socioeconomic status and the prevalence of fever among children under five. Using data from Ghana, Nigeria, Kenya and Sierra Leone, the study found that level of poverty (wealth status) influence the prevalence of fever among children under five in Africa.

Against the backdrop that fever is a major symptom of malaria, pneumonia, measles, diarrhoea etc, the control of these diseases in sub-Saharan Africa needs to take an integrated form, involving poverty reduction or welfare improvement. In the case of malaria, while provision of bed nets could be a crucial step in reducing the prevalence of fever, measures need to be put in place to ensure that recipients actually use the nets frequently and effectively. Such measures include hanging the nets for households as is currently being done by the National Malaria Control Programme in Ghana and community based education on effective use of bed nets.

Further, home management of fevers to reduce pneumonia and malaria cases should be adopted as an important step to the control of fevers. This is especially crucial in communities where access to health service is not available. Also intensifying vaccination campaigns in developing countries will go a long way to help curb the burden of fever through the control of its related diseases. In addition, general sanitation should be encouraged, especially among children. For instance, providing and promoting the use of improved toilet facilities in deprived communities and households. These recommendations are important to bridge the gap between poverty and the prevalence of fever as the poor can also access treatment and preventive measures.

## Competing Interests

The authors declare that they have no competing interests.

## Authors’ contributions

JN (1) and JN (2) conceived the study, undertook analysis and contributed to the write up of the manuscript. Both authors read and approved the final manuscript.
